# Metastatic renal cell carcinoma: the first report of unilateral fundus hemorrhage induced by sorafenib

**DOI:** 10.18632/oncotarget.9285

**Published:** 2016-05-11

**Authors:** Zhi Yong Li, Xin Xiang Fan, Yan Jun Wang, Kai Yao, Zhuo Wei Liu, Wen Tao Pan, Yun Lin Ye, Ping Yang, Yi Chuan Huang, Zhi Ming Wu, Fang Jian Zhou

**Affiliations:** ^1^ State Key Laboratory of Oncology in Southern China, Guangzhou, China; ^2^ Department of Urology, Sun Yat-Sen University Cancer Center, Guangzhou, China; ^3^ Collaborative Innovation Center for Cancer Medicine, Guangzhou, China; ^4^ Department of Urology, Sun Yat-Sen Memorial Hospital, Guangzhou, China

**Keywords:** RCC, sorafenib, TKIs, CRVO, unilateral

## Abstract

**Background:**

Renal cell carcinoma (RCC) is the most common type of kidney tumor with increasing incidence. Tyrosine Kinase Inhibitors (TKIs) are considered important treatment in the management of metastatic RCC. Some previous studies demonstrated that sorafenib treatment is associated with a significantly increased risk of potentially life-threatening adverse events, like bleeding. But bleeding at the fundus site is the rarest type of hemorrhage. As for TKIs' risk of bleeding, how we distinguish the degree of bleeding and what optimal strategies should we take to manage bleeding, needs to be studied systematically.

**Results:**

With a long-term exposure (17 months) to sorafenib, he experienced blurred vision in his right eye and was hospitalized. The patient's diagnosis was central retinal vein occlusion (CRVO) of the right eye. Unfortunately sorafenib was terminated.

**Materials and Methods:**

The authors describe the first case of unilateral fundus hemorrhage induced by sorafenib. A 42-year-old man was diagnosed metastatic left RCC, with clinical stage and prognostic risk being assessed as T4N1M1 and intermediate. He received a radical left nephrectomy and retroperitoneal lymph node dissection, with taking the oral multi-targeted TKI, sorafenib (800 mg daily) from 7 months to 7 days before the surgery and 7 days after the surgery restarting again until the occurrence of fundus hemorrhage.

**Conclusions:**

In this patient, long-term exposure to sorafenib possibly has increased the risk of fundus hemorrhage. This article provides us a previously undescribed morbidity associated with sorafenib, which reminds us of understanding the risk of bleeding and how this complication might be managed systematically.

## INTRODUCTION

RCC is the most common type of kidney tumor with increasing incidence. Recently it has been reported to be related with a rise in cancer-specific mortality in Western Europe and United States [1–2]. About 17% of patients with RCC are to be metastatic at initial examination [3] and lung parenchyma is the most common metastatic sites (50%–60%) [4]. For patients with metastatic RCC, the 5-year overall survival is less than 10% [5].

Interferon-α and interleukin-2 used to be the standard practice before 2006, which can only provide a modest survival advantage [6]. With the development of molecular-targeted therapies, TKIs such as sorafenib and sunitinib are considered important treatment in the management of metastatic RCC.

Generally, patients would keep on using sorafenib until severe side effect occurs or disease progression. But median exposure of 9–15 months is often recommended [7]. The longer administration of sorafenib, the greater of probability to develop cumulative toxicities will occur, so the patient could suffer from side effects. The most common reported sorafenib-related side effects include hand-foot syndrome (25%), rash/desquamation (27%), diarrhea (34%), anorexia (35%) and fatigue (40%) [8]. In addition, a previous meta-analysis of RCTs show us that the use of TKIs is related with an increased risk of fatal adverse effects, such as bleeding, congestive heart failure, arterial thrombotic events and so on [9]. As for too many possible side effects of sorafenib, especially for bleeding, understanding the risk of bleeding and how this complication might be managed systematically would be very important. It hasn't been discussed systematically before.

## RESULTS

### Case report

**Figure 1:**
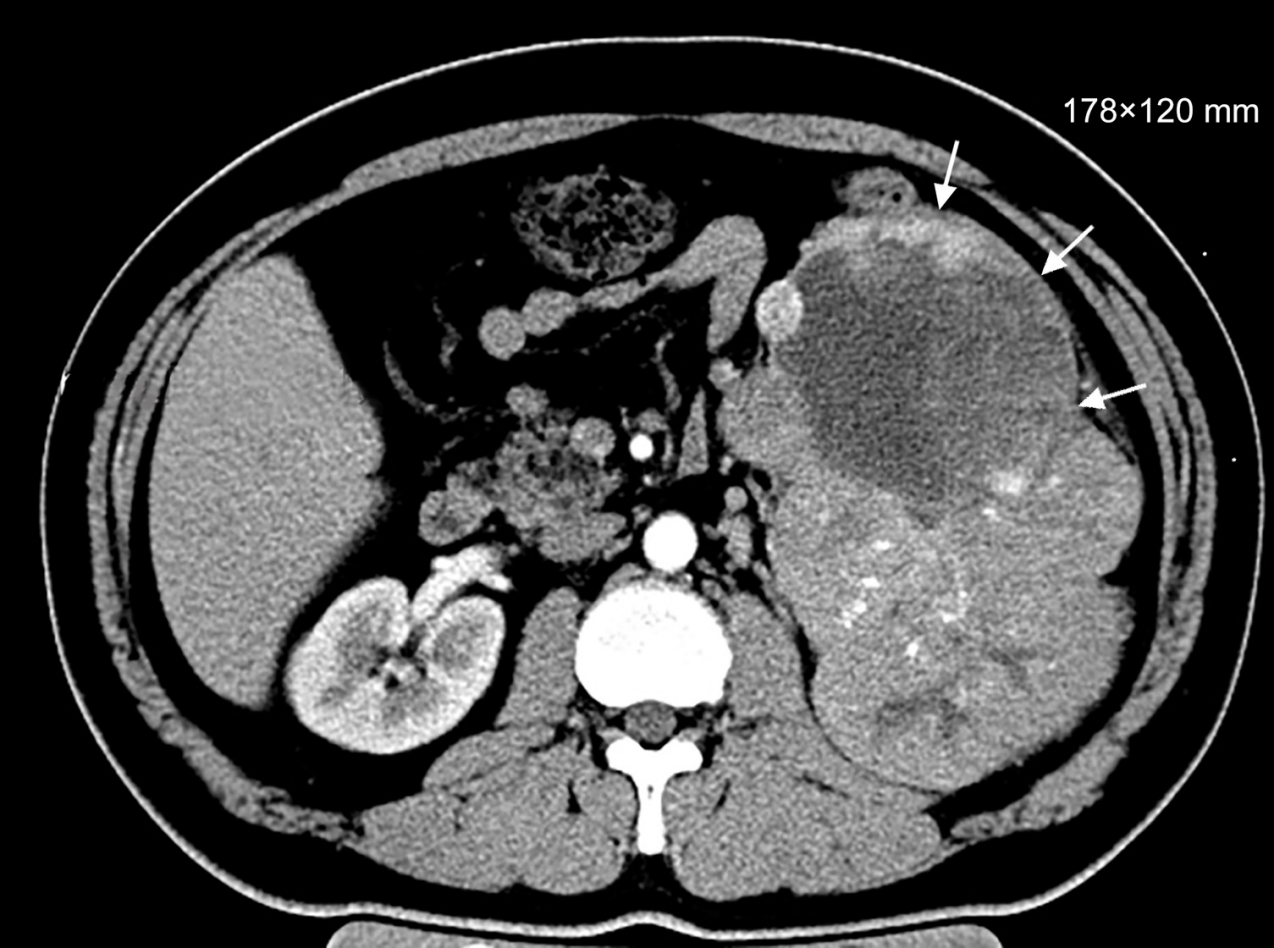
Renal CT (Without any treatment, November 2013)

A 42-year-old man complained of a single symptom of intermittent gross hematuria for one month and was first hospitalized in November 2013. He had no special medical and surgical history, no specific personal and family history. Radiological evaluation by a positron emission tomography/computed tomography (PET/CT) revealed massive accumulating spots in the lower-middle regions of the left kidney and, multiple lesions in the superior and middle lobes of the right lung and the inferior lobe of the left lung. Renal CT showed a soft tissue mass with a size of 178 × 120 mm in the lower-middle section of the left kidney (Figure 1). Based on radiological findings, the patient's clinical stage was assessed as T4N1M1.

### Risk assessing

Next, we analyzed the prognostic risk factors specified by the 2015 EAU (European Association of Urology) and 2016 NCCN (National comprehensive cancer network) Guidelines for Kidney Cancer:

- Corrected serum calcium level (Ca^2+^): 2.76 mmol/L
- Hemoglobin level: 134 g/L
- Lactate dehydrogenase (LDH): 156.4 U/L
- Interval from original diagnosis to the start of systemic therapy: less than one year.
- Karnofsky performance score: 90

According to the above criteria, the patient was assessed as being at intermediate risk.

### Diagnosis and treatment process

**Figure 2:**
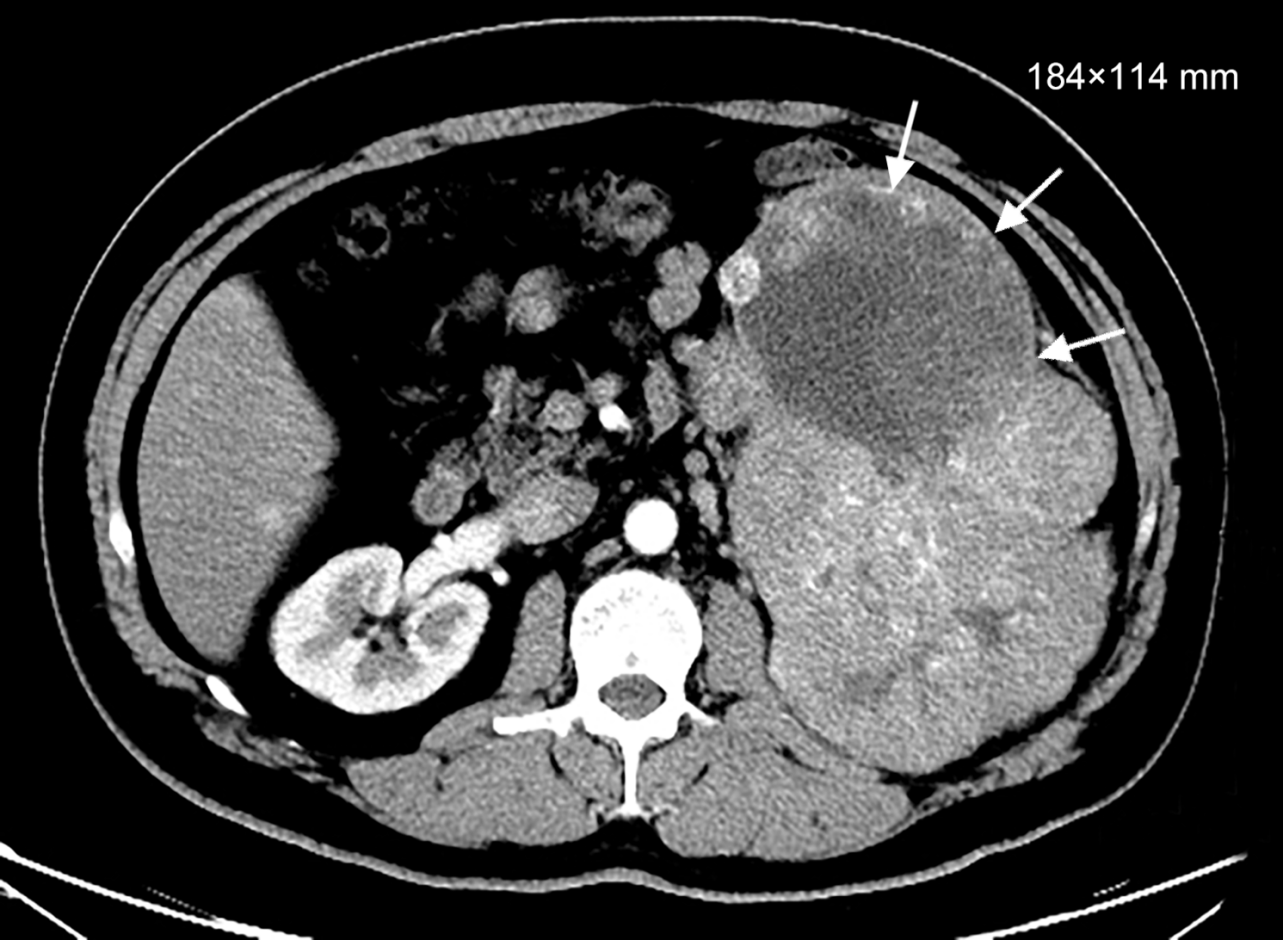
Renal CT (After treatment of sorafenib for 7 months, May 2014)

Because the tumor's large size prevented its direct removal, we treated the patient twice daily with 400 mg of sorafenib. After the first week of sorafenib administration, the hematuria disappeared and did not recur. However, after 4 weeks, several adverse effects appeared, including grade II diarrhea and grade III hand-foot syndrome. These side effects were well controlled by symptomatic treatment, such as the use of loperamide and topical emollient therapy, and the patient was able to continue sorafenib therapy. At 6-months of follow-up, total-body CT showed that no new lesions had developed and that partial remission of the pulmonary lesions had occurred. A large soft tissue mass with a size of 184 × 114 mm was found in the left kidney, indicating that the patient's disease was stable based on RECIST (Response Evaluation Criteria In Solid Tumors) guidelines (version 1.1), with an increase in total tumor size of less than 20% (Figure 2). The patient was then admitted and received a radical left nephrectomy and retroperitoneal lymph node dissection. As the pulmonary metastasis has been diagnosed, his operation was only a cytoreductive nephrectomy. 7 days before the surgery he stopped taking sorafenib and 7 days after the surgery he restarted agin. His pathological results revealed mixed renal cell carcinoma, with papillary renal cell carcinoma (60%), clear cell renal cell carcinoma (20%) and sarcomatoid carcinoma (20%). Furthermore, intravascular tumor thrombus was found, but renal pelvis, adrenal gland and lymph node haven't been invaded. His postoperative pathological stage was determined to be pT3aN0M1. After surgery, he underwent CT scanning every 6 months. These revealed the absence of the left kidney and stable disease status. Treatment with 400 mg of sorafenib twice daily was restarted 7 days after the operation until the occurrence of fundus hemorrhage 10 months after the operation.

### Prognosis

**Figure 3:**
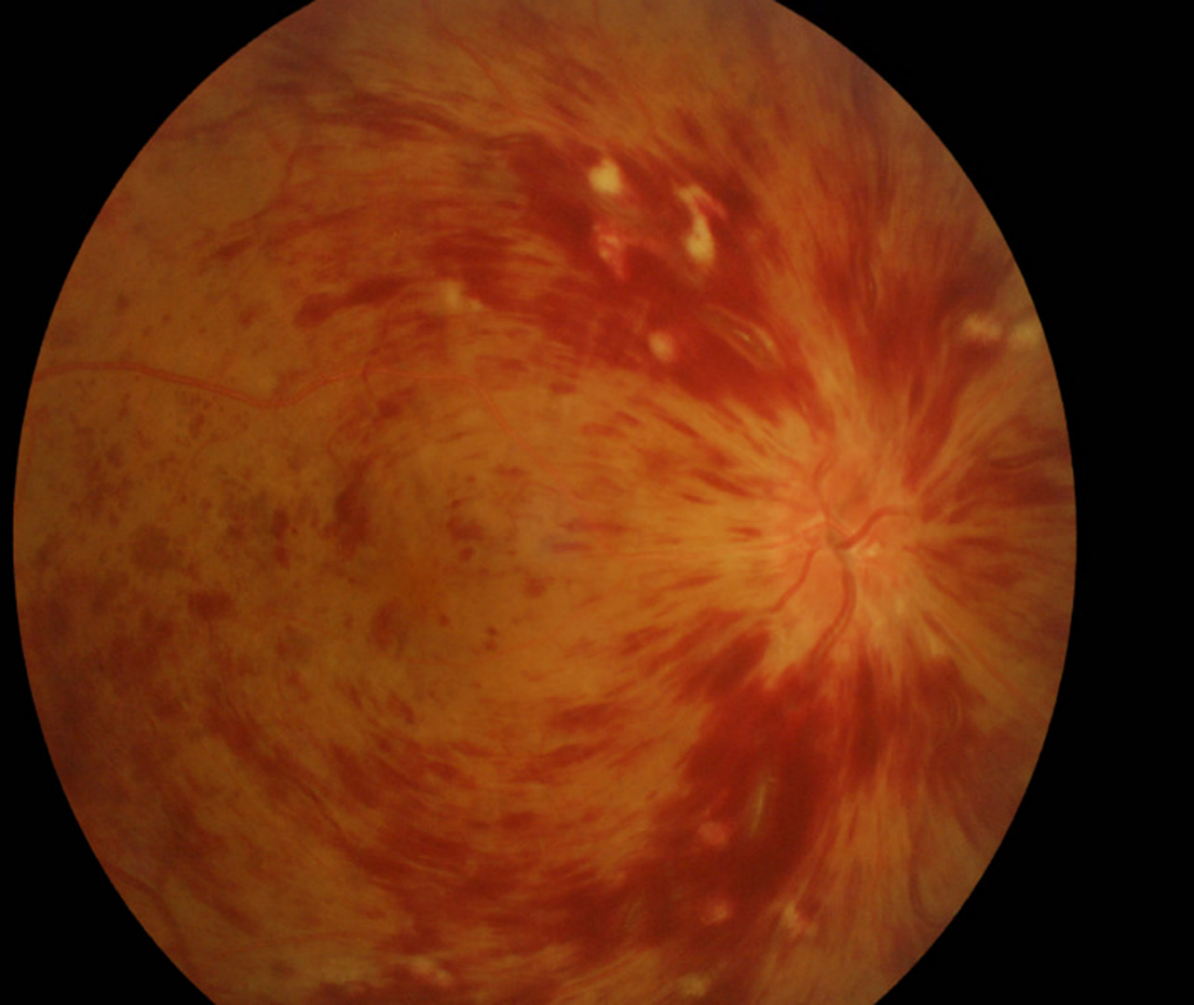
Fundus fluorescein angiography (fundus hemorrhage, March 2015)

Bleeding at the fundus site is the most severe and the rarest type of hemorrhage of the eye. After experiencing sudden blurred vision in his right eye 10 months after the surgery, the patient was hospitalized. On admission his best-corrected visual acuity of the right eye and left eye were 0.1 and 1.5 respectively. His right and left intraocular pressures were 13 mmHg and 14 mmHg, respectively. Moreover, his right and left ocular perfusion pressure (OPP) were normal: 53 mmHg and 52 mmHg, respectively. Additionally our patient had no special medical and surgical history, no specific personal and family history, and no previous history of abnormal fundus hemorrhage or bleeding disorders. The physical examination demonstrated obvious peripheral superficial hemorrhage, edematous optic disk, tortuous retinal veins and cotton wool spots in the macula in the right eye and no special physical sign in the left eye. His BMI was 27.46 kg/m^2^. His blood pressure was 130/82 mmHg, his blood glucose level was 4.58 mmol/L, and his triglycerides and cholesterol were 2.75 mmol/L and 5.63 mmol/L respectively, and no symptoms of ischemic heart disease were observed. Additionally, a carotid vascular ultrasound showed that the caliber of carotid arteries and the intima were normal. Moreover, laboratory findings, which included the results of immunological tests, a complete blood count, and examination of the coagulation routine, liver function and renal function, were normal. Fluorescein angiography (FFA) and optical coherence tomography (OCT) revealed hemorrhage around his right optic disk and posterior pole retina, and a thrombus in the central retinal vein in his right eye (Figure 3). The patient's diagnosis was CRVO of the right eye. He discontinued sorafenib and received treatment to prevent inflammation, protect the optic nerve (mecobalamine), and promote blood circulation. His vision gradually improved. His best-corrected visual acuity of the right eye and left eye were 0.3 and 1.5 respectively when leaving hospital. However, we still continue to be aware of the potential complication of retinal neovascularization after 12–16 weeks [10]. Laser photocoagulation will be administered if necessary. His vision sustainably improved. 3 weeks after his leaving hospital, his best-corrected visual acuity of the right eye and left eye were 0.4 and 1.5 respectively. Systemic therapy is necessary for our patient, thus we treated him with another oral multi-targeted TKI, axitinib (5 mg bid).

## DISCUSSION

TKI-induced bleeding was first reported by Hall et al. [11], who observed a case of epistaxis in a patient with RCC who was receiving sunitinib therapy. Later, in the largest study comparing sunitinib (*n* = 207) with placebo (*n* = 105), 14 patients (7%) in the sunitinib group experienced grade 1 or 2 epistaxis, whereas none of the patients in the placebo group developed epistaxis [12]. A meta-analysis by Youjin Je et al. yielding a total of 6779 patients demonstrated that the incidence of bleeding events (all grades) was 16.7% (95% CI 12.7–21.5) and that of high-grade events was 2.4% (1.6–3.9). The relative risk of all-grade bleeding events associated with sunitinib and sorafenib was 2.0 (1.14–3.49, *p* = 0.015). Additionally the authors found that the most common causes of all grade hemorrhagic events were hemoptysis (48.6%) and epistaxis (20.7%) [13]. Thus we learn that bleeding is a relatively common symptom in certain patients receiving TKI therapy. However, the extremely rare condition of hemorrhage at the unilateral fundus site has not previously been reported.

With respect to the treatment of metastatic RCC, four TKIs (sorafenib, sunitinib, axitinib and pazopanib) have been approved by the US Food and Drug Administration. All of these drugs affect antitumor and antiangiogenic activity [14]. They can inhibit vascular endothelial growth factor receptors (VEGFR1, VEGFR2 and VEGFR3), platelet-derived growth factor receptors(PDGFR α and PDGFR β), stem cell factor receptor (c-KIT), FMS-like tyrosine kinase 3 (FLT-3), colony-stimulating factor (CSF-1F) and the glial cell-line derived neurotrophic factor receptor (RET) [14–15]. Sorafenib has been shown to dramatically prolong progression-free survival compared with placebo in metastatic RCC [16].

Our patient had no previous history of abnormal fundus hemorrhage or bleeding disorders. After 17 months of treatment with sorafenib, blurred vision developed in his right eye. His diagnosis was CRVO of the right eye. He discontinued sorafenib and received treatment to prevent inflammation, protect the optic nerve (mecobalamine), and promote blood circulation. His vision gradually improved.

Mecobalamine, also called vitamin B12, is a water-soluble vitamin, playing a pivotal role in the normal functioning of nervous system and the formation of red blood cells. Its mechanism can be discussed as follows: two major coenzyme B12-dependent enzyme families, methylmalonyl Coenzyme A mutase (MUT) and 5-methyltetrahydrofolate-homocysteine methyltransferase (MTR), play a key role in functioning of B12. MUT can convert MM1-CoA to Su-CoA, which can extract energy from proteins and fats and help for proper myelin synthesis. MTR makes use of vitamin B12 to transfer a methyl group from 5-methyltetrahydrofolate to homocysteine, thereby generating tetrahydrofolate (THF) and methionine. THF plays an important role in DNA synthesis and results in effective production of cells with rapid turnover, especially red blood cells. All in all, mecobalamine is beneficial to nerve protection and acute blood loss, which is helpful to be used in our patient [17–18].

Common causes of fundus hemorrhage include: systemic diseases (hypertension, diabetes mellitus, hyperlipidemia, atherosclerosis associated diseases such as ischemic heart disease and stenosis or occlusion of the carotid arteries, systemic vasculitis associated with systemic lupus erythematosus, hematologic neoplasias such as leukemia, hypercoagulation diseases and drug therapy-induced diseases) and ocular diseases (glaucoma, ocular trauma, decreased OPP, external retrobulbar compression associated with an orbital neoplasma, and retinal arteriolar signs such as sclerosis of central retinal artery) [19]. These systemic diseases could all be excluded in the case of our patient. There were no signs of glaucoma or ocular trauma for our patient. When the fundus hemorrhage occurred, as it has been discuss above, his blood pressure, blood glucose, triglycerides, cholesterol, immunological tests, a complete blood count, coagulation routine, liver function, renal function, intraocular pressures and OPPS were all normal, and no symptoms of ischemic heart disease were observed. FFA and OCT indicated no external retrobulbar compression and no sclerosis of the central retinal artery. After eliminating all the potential causes listed above, we concluded that long-term exposure to sorafenib possibly caused the fundus hemorrhage. According to a previous Phase II clinical study assessing sorafenib in patients with gastrointestinal stromal tumors, among all the reported adverse events thrombosis occupy 6% [20]. In addition, as for a previous case report by Szymon Szczepanik et al. different from us showed that a 73-year-old man with disseminated metastatic renal cell carcinoma suffered from bilateral central retinal vein occlusion. He had been on treatment of sorafenib for 13 months [21]. Although bilateral CRVO has been reported in 1–14% of patients, it is typically unilateral [22]. Because the central retinal veins in the two eyes have different tolerances, fundus hemorrhage most commonly occurs in a single eye. Indeed, CRVO leading to fundus hemorrhage occurred only in our patient's right eye.

**Figure 4:**
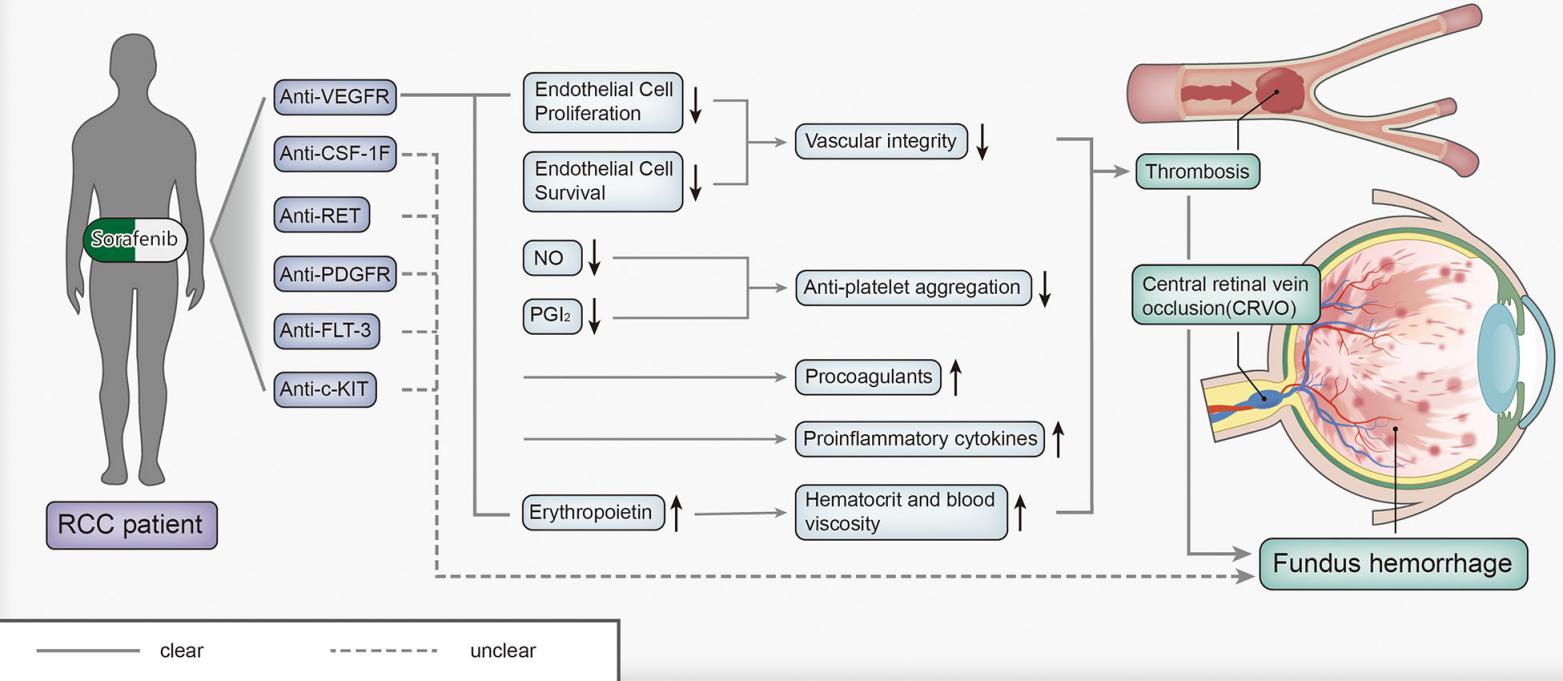
The pathogenesis of fundus hemorrhage

A meta-analysis by Nalluri et al. indicated that administration of bevacizumab (an angiogenesis inhibitor) may be associated with an increased likelihood of venous thromboembolism [23]. CRVO is a type of venous thromboembolism that can lead to fundus hemorrhage. The pathogenesis of this phenomenon may involve the following steps (Figure 4): First, the development of CRVO may stem from the anti-VEGFR effect of TKIs. We know that VEGF plays a key role in endothelial cell proliferation and, endothelial cell survival; thus, the Inhibition of VEGF by TKI treatment could jeopardize vascular integrity [24], ultimately resulting in thrombosis. In addition, sorafenib may not only stimulate the release of procoagulants from a tumor into the blood stream, but also promote the expression of proinflammatory cytokines, which lead to vascular damage and *in situ* thrombus formation [25]. Moreover, inhibition of VEGF could increase hematocrit and blood viscosity by inducing overproduction of erythropoietin, which could increase the risk of thrombosis. Furthermore, VEGF may increase the production of nitric oxide (NO) and prostacylin (PGI_2_), which could inhibit platelet aggregation [26]. Inhibition of VEGF causes decreases in NO and PGI_2_, increasing the potential for the development of thrombosis. After considering all the above-mentioned contributors to the pathogenic process, we speculated that administration of sorafenib may be related to the risk of developing thrombosis. Unfortunately, in our patient, thromboembolism appeared in the central retinal vein, causing his fundus hemorrhage, an extremely rare condition. Sorafenib targets multiple molecules, the question of, whether non-VEGFR targets, such as PDGFRs, RET, FLT-3, c-KIT and CSF-1F, play a role in the mechanism of fundus hemorrhage remains unresolved and requires additional investigation.

With respect to the occurrence of fundus hemorrhage after TKI therapy, age, obesity, cigarette smoking, and aforementioned systemic and ocular causes are considered risk factors [27]. Therefore, for patients treated with sorafenib who exhibit these risk factors, physicians should be aware of the possibility of fundus hemorrhage.

To our knowledge, this case we report here is the first described instance of sorafenib-induced unilateral fundus hemorrhage. The previous statistics showed us that the administration of sorafenib was associated with significantly increased risk of bleeding. In this patient, long-term exposure to sorafenib possibly has increased the risk of fundus hemorrhage. In spite of our current findings, widespread use and beneficial outcome of sorafenib in the treatment of RCC, HCC or GI stromal tumor, sorafenib should be continuously offered to these patients. However, doctors must be aware of the potential for fundus hemorrhage. Early detection, discontinuation of sorafenib and administration of symptomatic treatment as rapidly as possible could help prevent fundus hemorrhage. Fundus hemorrhage requires immediate treatment because it is associated with a high probability of vision loss. In cases of fundus hemorrhage, other potential causes should be eliminated before diagnosing the condition as sorafenib-induced hemorrhage.

We hope that our study helps to arouse awareness of the risk of ophthalmologic complications, looking for the potential symptoms, signs and physical exam findings of fundus hemorrhage when taking TKIs. Ulteriorly we hope to promote further study of risk factors, prevention, treatment and how long TKIs should be used.

## MATERIALS AND METHODS

### Laboratory examination

Processing and laboratory analyses of blood sample were performed in the laboratory at the Sun Yat-sen University Cancer Center which operates under the principles of Good Laboratory Practice. It has SOP for sample receipt, processing, and analysis.

### Imaging

A PHILIPS multidetector 64-section CT system (Netherlands) was used for imaging. Parameters were as follows: 5 mm section thickness, 5 mm intersection gap, matrix 512 × 512 and field of view: 20 × 20 cm^2^−40 × 40 cm^2^. As the contrast agent Iohexol was used at a dose of 2 ml/kg. The injection flow rate was 3 ml/s. Arterial phase scanning appeared 20–30s after the injection. Venous phase scanning was emerged about 60 s after injection. In addition, delayed phase scanning showed up 4 mins after injection. The coronal and sagittal planes with a 5 mm section thickness and a 5 mm interval were reformatted.

### Optic examination statement

Fundus fluorescein angiography (FFA) and optical coherence tomography (OCT) were used for optic examination statement. For FFA, sodium fluorescein as the contrast agent was injected into forearm veins within 3–5s. The amount was 5 milliliter. After 15s the optic shooting photo was performed. For OCT, uses light to capture micrometer-resolution, three-dimensional images from within optical scattering media. Its longitudinal resolution was 14 μm and longitudinal speed was 160 mm/s.

### Study procedures

After experiencing blurred vision in his right eye, the patient was hospitalized. Upon admission, he was examined via FFA and OCT, which detected hemorrhage around the right optic disk and posterior pole retina. After eliminating the common causes of fundus hemorrhage (systemic and ocular diseases), the diagnosis was central retinal vein occlusion (CRVO) of the right eye. His treatment entailed discontinuing sorafenib, and receiving therapy to prevent inflammation, protect the optic nerve (mecobalamine), and promote blood circulation. Blood sample and CT were performed in Sun Yat-sen University cancer center.
